# Linkers: An Assurance for Controlled Delivery of Antibody-Drug Conjugate

**DOI:** 10.3390/pharmaceutics14020396

**Published:** 2022-02-11

**Authors:** Rotimi Sheyi, Beatriz G. de la Torre, Fernando Albericio

**Affiliations:** 1 School of Chemistry and Physics, University of KwaZulu-Natal, Durban 4001, South Africa; ebenrotex4fun@gmail.com; 2 Kwazulu-Natal Research Innovation and Sequencing Platform (KRISP), College of Health Sciences, University of KwaZulu-Natal, Durban 4001, South Africa; 3 Institute for Advanced Chemistry of Catalonia (IQAC-CSIC), 08034 Barcelona, Spain; 4 Networking Centre on Bioengineering, Biomaterials and Nanomedicine (CIBER-BBN), Department of Organic Chemistry, University of Barcelona, 08028 Barcelona, Spain

**Keywords:** antibody-drug conjugates (ADCs), bioconjugation, chemotherapy, cytotoxic drug, FDA, monoclonal antibody, linker, tumor

## Abstract

As one of the major therapeutic options for cancer treatment, chemotherapy has limited selectivity against cancer cells. Consequently, this therapeutic strategy offers a small therapeutic window with potentially high toxicity and thus limited efficacy of doses that can be tolerated by patients. Antibody-drug conjugates (ADCs) are an emerging class of anti-cancer therapeutic drugs that can deliver highly cytotoxic molecules directly to cancer cells. To date, twelve ADCs have received market approval, with several others in clinical stages. ADCs have become a powerful class of therapeutic agents in oncology and hematology. ADCs consist of recombinant monoclonal antibodies that are covalently bound to cytotoxic chemicals via synthetic linkers. The linker has a key role in ADC outcomes because its characteristics substantially impact the therapeutic index efficacy and pharmacokinetics of these drugs. Stable linkers and ADCs can maintain antibody concentration in blood circulation, and they do not release the cytotoxic drug before it reaches its target, thus resulting in minimum off-target effects. The linkers used in ADC development can be classified as cleavable and non-cleavable. The former, in turn, can be grouped into three types: hydrazone, disulfide, or peptide linkers. In this review, we highlight the various linkers used in ADC development and their design strategy, release mechanisms, and future perspectives.

## 1. Introduction

Cancer is a serious life-threatening disease [1] causing over 8 million deaths worldwide each year [2]. This disease is commonly treated by surgery [3], radiotherapy [4] and chemotherapy [5,6], the latter being the treatment most widely used due to its capacity to target rapidly dividing cancer cells [2]. However, the use of chemotherapeutic drugs faces constant limitations in terms of non-specificity, meaning that they kill not only the tumor cells but also healthy cells and cause serious adverse reactions, narrow therapeutic windows, and increased drug resistance [7,8,9,10,11,12,13]. Targeted drug therapy could potentially address these challenges, as it facilitates the delivery of drug agents to unhealthy cells without harming healthy ones [14,15,16,17,18,19,20]. Antibodies are a rapidly growing class of drug that play a major role in human health, mostly in oncology, autoimmunity and chronic inflammatory diseases [21,22,23].

**Figure 1:**
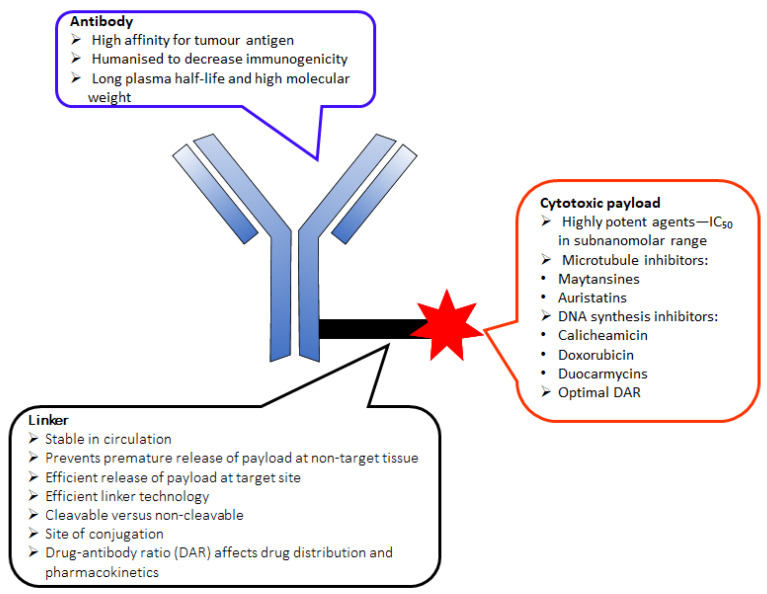
Schematic diagram of an antibody-drug conjugate (ADC).

The development of antibody drug conjugates (ADCs) as targeted drug therapies has made significant progress over the last century [24,25,26,27]. In 1897, Paul Ehrlich, a German scientist, was the first to propose the ‘magic bullet’ theory for delivering toxic compounds to unhealthy cells. His idea later evolved to become what is today known as ADCs [28,29,30,31]. This class of therapeutic agents consists of recombinant monoclonal antibodies (mAbs) (which direct the drug to the target cells) that are covalently bound to cytotoxic chemicals (known as warheads) via synthetic linkers, as shown in Figure 1. ADCs offer the prospect of delivering a toxic payload directly to a target, with minimal off-target toxicity [32].

Two main factors determine the successful development of an ADC. First, the choice of linker is crucial, as it accounts for: (i) high plasma stability in circulation to prevent premature drug release (t_1/2_ > 1 week); (ii) maintenance of the properties of the mAb and cell-killing ability of the cytotoxic drugs, along with a reduction of systemic toxicity; (iii) high aqueous solubility that allows bioconjugation of lipophilic drugs and prevents antibody aggregation; and (iv) drug release in the right circumstances to maximize the therapeutic effect [33,34,35,36,37,38]. Second, successful ADC development is also dependent on the drug-antibody ratio (DAR), which is the number of drug molecules attached to the antibody via a linker. A low DAR decreases ADC efficacy, while a high DAR often results in ADC instability, increased systemic effect, and reduced half-life, and it alters the pharmacokinetic properties of the molecule [26]. However, the choice of appropriate linker depends on the functional groups present in the mAbs and cytotoxic drugs. This review provides an update of FDA-approved ADCs and the linkers used in the design of these drugs.

## 2. The Key Components of an ADC

### 2.1. Monoclonal Antibody (mAb)

An important component of an ADC is the antibody. The basic premise for the selection of an antibody for ADC design is its ability to specifically identify and bind to a well-characterized tumor antigen–receptor and deliver the payload to the tumor cell in the process. The antibody must also have high binding affinity to the specific target antigen, low immunogenicity, appreciable stability in the bloodstream, and low cross-reactivity [2,35]. Most antibodies used in ADC design are selected from human immunoglobulin G (IgG) subclasses (IgG1, IgG2, IgG4), which consist of tw heavy and two light chains [20,26,33]. ADC-targeted antigens must be highly expressed on the tumor cells [39] and should also have internalization properties to enhance the receptor-mediated endocytosis of the ADC [40]. Currently, Nectin4, CD79b, CD22, CD33, HER2, CD30, FOLR1, and TROP2 are the most targeted antigens in ADCs. In addition, over 70 other antigens are in different stages of clinical development [41,42,43,44].

### 2.2. Cytotoxic Drug

**Figure 2:**
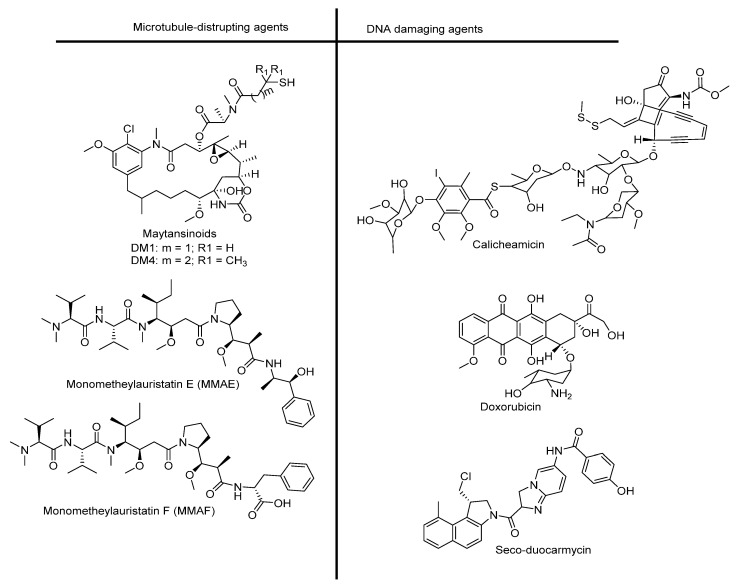
Cytotoxic drugs used in ADC design.

Cytotoxic drugs are highly potent agents used to kill cancer cells. They can prevent cell division either by disrupting microtubule assembly, thus inhibiting mitosis, or by binding to the minor groove of DNA, leading to the cleavage of double-strand DNA. The latter process causes cell death/apoptosis [45,46]. Therefore, a cytotoxin is required to have maximum plasma stability and an in vitro subnanomolar IC_50_ (half maximal inhibitory concentration) value for tumor cells, as only 1–2% of injected ADCs reach the tumor [33,47]. Cytotoxic drugs are transported in the bloodstream throughout the body. At present, auristatins and maytansinoids are the drugs most frequently used for ADC development (see Figure 2 for their chemical structures) [37]. The conjugation of these drugs to mAbs is usually achieved via a chemical linker attached to the thiol residue of Cys or to the amino group of Lys antibody molecule. Several drugs used in the design of ADCs are listed in Figure 2.

## 3. Linker Chemistry and Conjugation to Antibody

The linker, which is the focus of this review, is an essential component in ADC design. It connects the antibody to the cytotoxic payload via covalent conjugation [26,48,49,50]. The key requirement of a linker is that it must ensure chemical stability of the ADC within the bloodstream (i.e., have a half-life 10 times longer than the ADC) and allow for rapid release of the payload at the target site after internalization [51,52]. In addition to the above parameters that minimize premature drug release [39], hydro/lipophilicity, a property that enhances the coupling of payloads and reduces immunogenicity, is also a key aspect of linkers [53,54].

**Figure 3:**
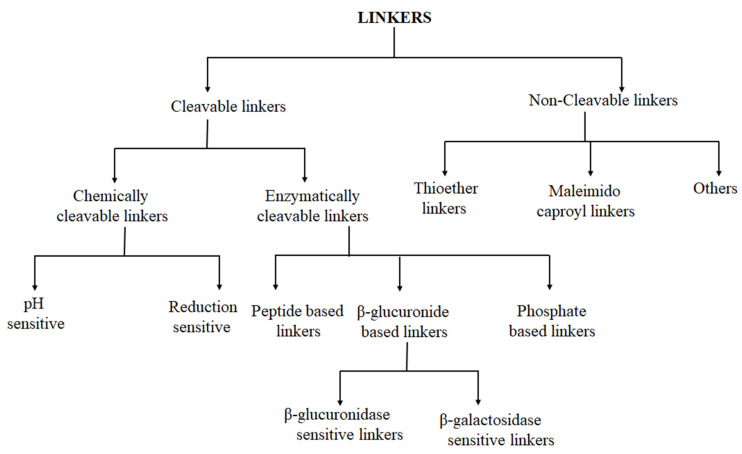
Classification of linkers in ADCs.

Two types of linkers, namely cleavable and non-cleavable, are used in ADC development (Figure 3). These linkers play major roles in determining pharmacokinetic properties, selectivity, therapeutic index, and the overall success of the ADC. With the development of ADCs, a series of linkers have been exploited [37]. Cleavable and non-cleavable linkers have been proven to be safe in preclinical and clinical trials. Linkers are broadly classified on the basis of the drug release mechanism and their stability in circulation [55,56].

### 3.1. Cleavable Linkers

Cleavable linkers play a pivotal role in the success of ADCs. They are stable in blood circulation for a long time and efficiently release their payload in the tumor microenvironment. Some of the cleavable linker strategies available and cleavage conditions are summarized in Table 1 and are subsequently discussed.

#### 3.1.1. Chemically Labile Linkers

ADCs containing chemically labile linkers, including acid-cleavable linkers and reducible linkers, take advantage of the differential properties between plasma and the cytoplasmic compartments to release the payload.

##### Acid-Cleavable/pH-Sensitive Linkers

This class of linkers is stable to alkaline environments but highly sensitive to acidic environments such as the hydrazone. They take advantage of the low pH of the endosome (pH = 5–6) and the lysosome (pH = 4.8) to trigger the hydrolysis of the acid-labile hydrazone linker and subsequently release the payload [48].

**Figure 4:**
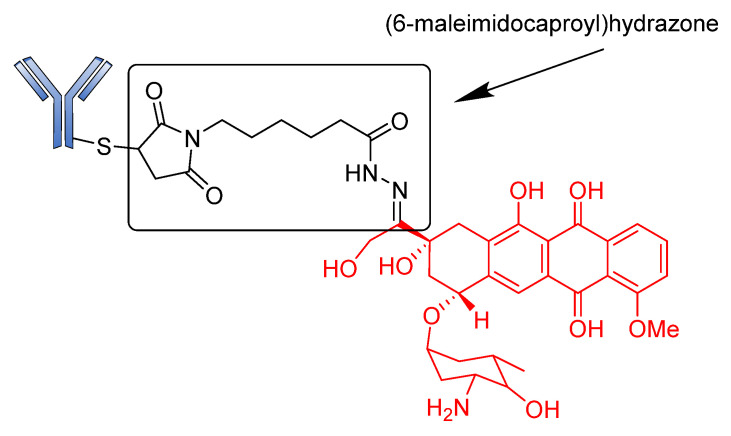
Structure of BR96-doxorubicin.

BR96-Doxorobicin (BR96-Dox) is a good example of a drug conjugate constructed using an acid-sensitive linker. This ADC was designed by linking doxorubicin, an intercalating agent that blocks DNA replication over a (6-maleimidocaproyl) hydrazone linker bonded to cysteine residues of humanized BR96 monoclonal antibody (Figure 4) [74]. The preclinical results of BR96-Dox were remarkable due to its potential to deliver high doses of doxorubicin to tumors. These doses were found to be capable of curing hypodermic human breast, and lung and colon tumors [75,76]. Although BR96-Dox has a high DAR, it showed insufficient potency in clinical trials. In addition, the half-life of the drug was too short compared to that of the naked BR96 mAb in humans [77].

**Figure 5:**
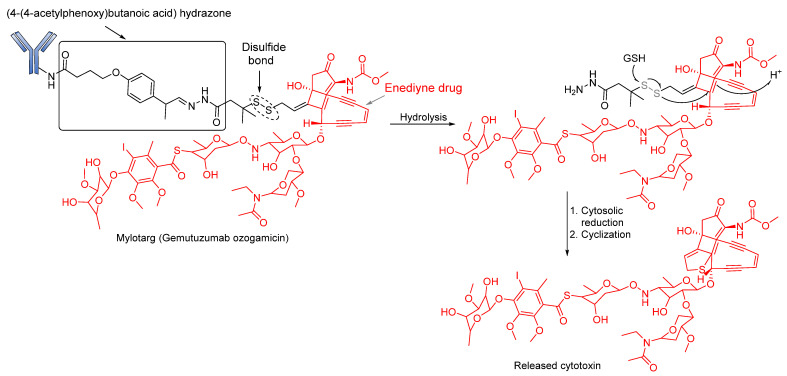
Cleavage mode of hydrazone linker in Mylotarg. Under strong acidic conditions, the metabolite—calicheamicin—is released first by hydrolysis of the hydrazone moiety, followed by the cytosolic reduction of the disulfide bond to afford the free sulfide anion. This then forms a thiophen ring through cyclization.

Hydrazone linkers were also explored in the design of Gemtuzumab ozogamicin (Mylotarg) and Inotuzumab ozogamicin (InO; Besponsa). Mylotarg, the first ADC to gain regulatory approval, is used for the treatment of patients suffering from relapsed acute myelocytic leukemia (AML). This ADC consists of *N*-acetyl-γ-calicheamicin (a DNA- damaging agent) covalently attached to humanized anti-CD33 antibody (hP67.6) through an acid-cleavable hydrazone linker [78]. The 4-(4-acetylphenoxy) butanoic acid moiety permits conjugation to Lys residues of the hP67.6 antibody over an amide bond and forms an acyl hydrazone linkage with *N*-acetyl-γ-calicheamicin dimethyl hydrazide. Upon internalization of the ADC, the calicheamicin prodrug is released by hydrolysis of the hydrazone into the lysosomal compartment of CD33-positive tumor cells. The DNA-damaging agent (enediyne drug) is then activated by reduction of the disulfide bond (Figure 5). However, the stability of this bond has been enhanced by the introduction of two methyl groups to the α-carbon bearing the disulfide bonds to prevent premature release of the calicheamicin metabolite [77]. Initial clinical studies of Mylotarg in relapsed patients led to its approval in 2000. However, the instability of the linker and heterogeneous nature of the conjugate caused the premature release of the drug before it reached its target site [79] and, consequently, the ADC was voluntarily withdrawn from the market by the US FDA in 2010 [80]. However, this ADC was later reapproved for use in 2017 when its benefits were considered to outweigh its risks [19].

Similarly, collaborative work between Wyeth and Celltech led to the development of Inotuzumab ozogamicin (CMC-544, Besponsa), a calicheamicin-based ADC that consists of a recombinant humanized anti-CD22 antibody attached to *N*-acetyl-γ-calicheamicin dimethyl hydrazide via the acid-labile (4-(4′-acetylphenoxy)butanoic acid) hydrazone linker. Although this ADC is closely related to Mylotarg, it showed more stability in both human plasma and serum (rate of hydrolysis of 1.5–2%/day over 4 days) [81,82]. Used to treat relapsed B-cell precursor acute lymphoblastic leukemia, Besponsa differs from Mylotarg in that it targets CD22-bearing antigen cells.

##### Reducible Linkers

Reducible or glutathione-sensitive disulfide linkers are alternatives to acid-labile hydrazone linkers in ADC design. Disulfide bonds are comparatively stable in circulation yet are reductively cleaved by intracellular glutathione to release the payload [26]. Glutathione is a low molecular weight thiol present in the cytoplasm (1–10 mmol/L) and extracellular environment (2–20 µmol/L in plasma) [6,20,83,84]. The basic premise for the cleavage of ADCs containing these linkers is the difference in reduction potential in the intracellular compartment as opposed to plasma, bearing in mind that glutathione is highly released during cell replication; hence, a high concentration of glutathione can be found in cancer cells [85]. These linkers generate a neutral payload that can diffuse into neighboring cancer cells and produce bystander-killing effects [86].

As earlier discussed, disulfide linkers have found significant clinical applications from their combination with hydrazone in the development of Pfizer’s Mylotarg and Besponsa. Remarkably, Immunogen reported the fortuitous development of some disulfide-based ADCs: SAR3419 (antiCD19 maytansine conjugate), IMGN901 (anti-CD56 maytansine conjugate), and AVE9633 (anti-CD33 maytansine conjugate).

**Figure 6:**
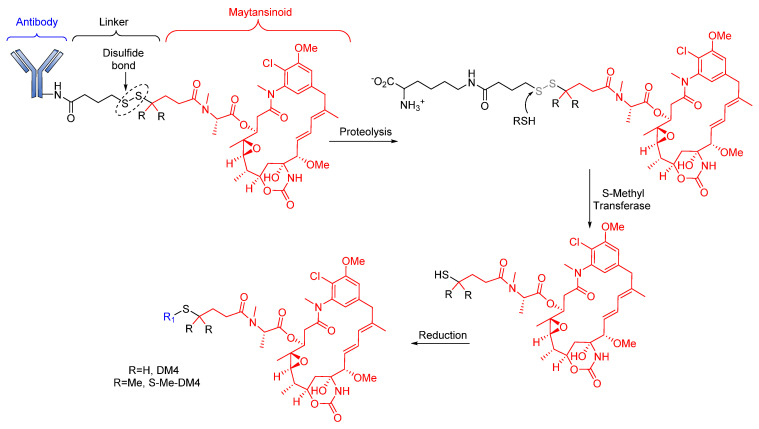
Cleavage mode of disulfide linker in huC242-SPDB-DM4. The ADC loses antibodies by proteolysis and then undergoes disulfide bond cleavage to form the active drug. The drug is then metabolized with S methyl transferase.

Notably, in 2011, Kellogg et al. reported the synthesis of huC242-SPDB-DM4 (IMGN242), a disulfide-containing ADC [87], comprises huC242 antibody linked to tubulin. This ADC inhibits cytotoxin (maytansinoids, DM4) via a disulfide linker with varying levels of steric hindrance around the disulfide (Figure 6) [87]. Unlike its non-cleavable counterpart (huC242-SMCC-DM1), huC242-SPDB-DM4 has a DAR > 4, conferring it with higher in vitro activity. The study of this ADC also revealed its high stability to dithiothreitol reduction in isolated plasma of CD1 mice. This drug conjugate, with intermediate disulfide bond stability and two methyl groups on the maytansinoid side of the bond and no methyl groups on the linker side of the bond, showed a better efficacy than huC242-SMCC-DM1 [87]. An in vivo study of this maytansinoid conjugate also revealed its effectiveness in killing neighboring non-targeted cancer cells as a result of on-target cleavage of the cytotoxic metabolites, which diffuse into neighboring cells to elicit a bystander effect.

**Figure 7:**
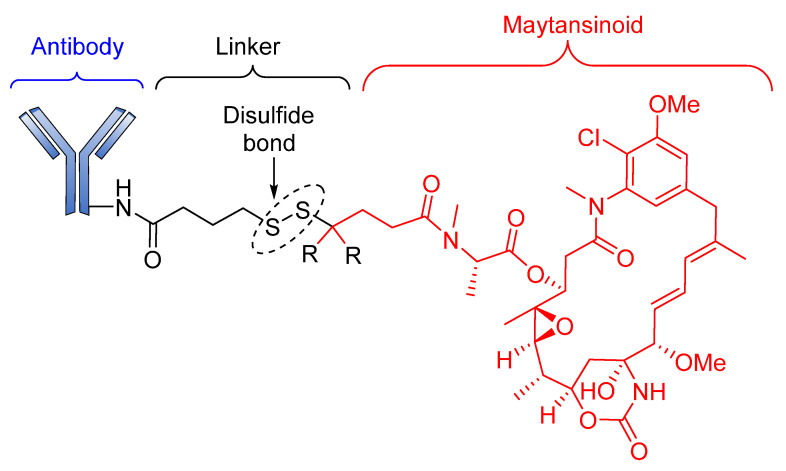
The structure of IMGN901.

Although Lorvotuzumab mertansine (IMGN901) is still in phase II clinical trials, researchers at Immunogen have reported an anti-CD56 effect. IMGN901 consists of a potent maytansinoid attached to a CD56-binding monoclonal antibody through a disulfide linker (Figure 7) [88].

#### 3.1.2. Enzyme-Cleavable Linkers

Chemically labile linkers often have limited plasma stability, thereby leading to premature drug release. In this regard, as an alternative strategy, enzyme-cleavable linkers have achieved clinical success in controlled drug release. Unlike chemically labile linkers, enzyme-cleavable ones take advantage of the high concentration of unique hydrolytic enzymes in cellular compartments to degrade peptides and carbohydrates.

##### Peptide-Based Linkers

**Figure 8:**
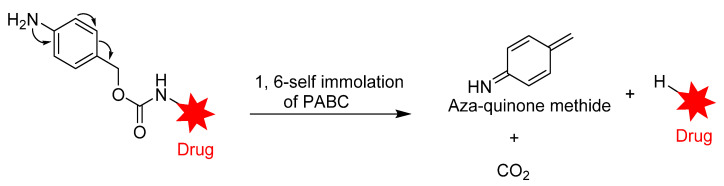
Straightforward cleavage mechanism of p-aminobenzyl carbamate (PABC) containing conjugate.

Peptide-based linkers, also known as lysosomal protease-sensitive linkers, such as valine–citrulline (Val–Cit), phenylalanine–lysine (Phe–Lys), and valine–alanine (Val–Ala) dipeptide linkers, are the most widely used linkers in ADC design. This strategy utilizes intracellular protease, such as Cathepsin B, which recognizes and cleaves a dipeptide bond, thus leading to the release of the cytotoxic drugs [89]. Due to unsuitable pH conditions and serum protease inhibitors, peptide-based linkers show greater systemic stability, with rapid enzymatic release of the payload in the target cell [90]. Exploring these types of linkers in ADC development often requires a conjugating spacer molecule due to the bulky nature of payload. The reagent most commonly used for this purpose is para-aminobenzyl carbamate (PABC) (Figure 8), which shows self-cleavage capacity, thereby facilitating the release of the unmodified payload [70].

**Figure 9:**
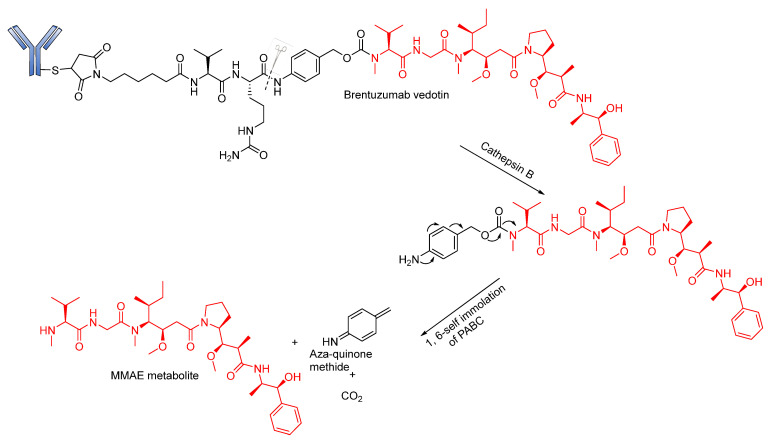
Cleavage mode of Brentuzumab vedotin.

Developed by Seattle Genetics/Millennium, FDA-approved Brentuximab vedotin (BV; Adcetris) is a good example of an ADC in which this linker has been explored with marked clinical success. BV consists of MMAE conjugated to an anti-CD30 antibody via a self- immolative protease (cathepsin B-sensitive) Val–Cit- para-aminobenzyl carbamate (PABC) linker [20,55]. BV is internalized into CD30-expressing cells, and the dipeptide bond undergoes proteolytic cleavage followed by self-immolation via a 1,6 elimination of PABC to release MMAE (Figure 9). Although ADCs containing this linker are generally stable in physiological conditions, an unidentified serine protease is known to cleave the linker in mouse plasma [20,91]. BV gained accelerated approval in 2011; however, due to several adverse effects (neuropathy, neutropenia, anemia and thrombocytopenia), its approval was halted [92,93]. However, after a series of modifications, it was fully approved in 2015, and it is considered suitable for the treatment of Hodgkin’s lymphoma [94,95].

**Figure 10:**
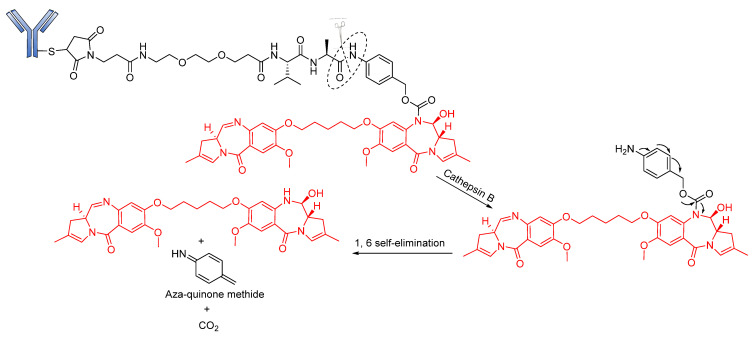
Cleavage mode of Val–Ala-containing Rovalpituzumab tesirine.

An alternative to the Val–Cit linker is the protease-cleavable Val–Ala dipeptide linker, which is being used in the development of many pyrrolobenzodiazepine-containing ADCs. Currently in phase III clinical trials, Rovalpituzumab tesirine (Rova-T; SC16LD6.5) is a biomarker-specific ADC for the exclusive targeting small-cell lung cancers expressing Delta-like protein 3 (DLL3) antigen [96]. Rova-T is made up of SC16 antibody conjugated to a pyrrolobenzodiazepine (PBD) payload via a PEG8 spacer, namely a Val–Ala linker. However, the bulky nature of the drug calls for the use of a self-immolative PABC to afford the straightforward release of the payload. Similar to BV, Rova-T first undergoes proteolytic cleavage of the Val–Ala linker followed by self-immolation of PABC to release the PBD payload (Figure 10) [97].

##### Glycosidase-Sensitive Linkers

β-Glucuronidase-cleavable linkers

Glycosidases, such as β-glucuronidases, are a class of hydrolytic lysosomal enzymes that degrade β-glucuronic acid residues into polysaccharides. They are found exclusively in the lysosomal compartment of the cell, and they work under hydrophilic environments to release payloads from conjugates. Similar to cathepsin B, β-glucuronidases are secreted in the necrotic areas of some tumors. Remarkably, these molecules are enzymatically active in the extracellular environment. Given this property, in 1988, Tietze et al. addressed for the first time the activity of β-glucuronidase-responsive prodrugs on neighboring tumor cells [69].

**Figure 11:**
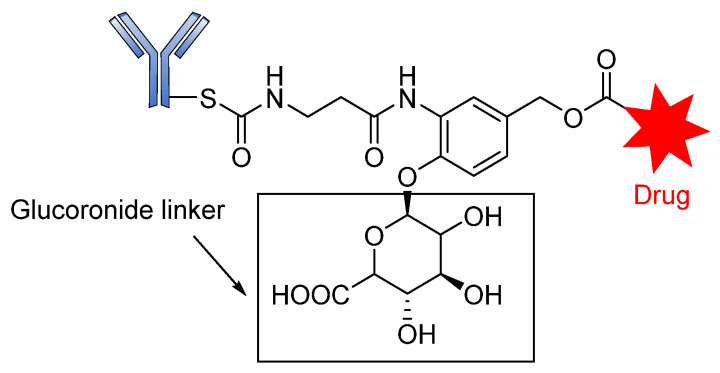
The structure of an ADC containing β-glucuronic acid.

Moreover, in a comprehensive study, Jeffrey and coworkers evaluated the properties of β-glucuronic-based acid linkers ADCs. Using highly potent microtube inhibitors (MMAE and MMAF) and a DNA-damaging agent (doxorubicin), β-glucuronidase-susceptible ADCs were designed by covalent conjugation of the cytotoxic agents to the antibody via a β-glucuronide linker attached to a self-immolative PABC spacer molecule (Figure 11) [98].

**Figure 12:**
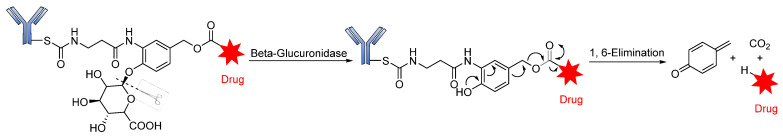
The mechanism by which an ADC containing β-glucuronic acid is released.

The introduction of the self-immolative spacer enhances linker stability and allows safe release of the potent cytotoxin [99]. The resultant β-glucuronide MMAF drug conjugates gave a DAR of 8.3 with low levels of aggregation and a half-life of 81 days, far greater than that of the dipeptide linkers. Apart from the use of auristatins and doxorubicin, this strategy has also been employed in targeting special classes of cytotoxic agents such as anthracyclines, camptothecin derivatives, taxanes, hedgehog inhibitors, nitrogen mustards, and histone deacetylase inhibitors [69]. Most of these drug conjugates contain the self- immolative PABC spacer molecule between the β-glucuronide linker and the drug moiety, thereby offering straightforward release of the payloads upon internalization [69]. The release of the drug therefore proceeds in two steps: (i) the enzymatic hydrolysis of the glycosidic bound; and (ii) the spontaneous decomposition of the linker, leading to the release of the active compound (Figure 12) [70].

β-Galactosidase-cleavable linkers

**Figure 13:**
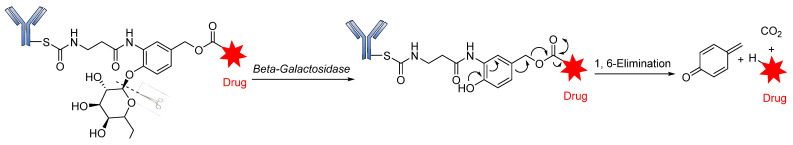
The mechanism by which an ADC containing β-glucuronic acid is released.

Another class of hydrolytic lysosomal enzymes is β-galactosidase, which degrades β-glycosidic linkage formed between a galactose and its organic moiety. Kolodych and coworkers recently described both the in vitro and in vivo activities of β-galactosidase-cleavable ADCs. The study revealed that galactosidase-based drug conjugates have greater therapeutic efficacy in isolated mouse plasma compared to the approved Trastuzumab emtansine used for the treatment of breast cancer [100]. Similar to β-glucuronidase, β-galactosidase is overexpressed in certain tumors, where it hydrolyzes β-galactoside (Figure 13).

##### Phosphatase-Cleavable Linkers

**Figure 14:**
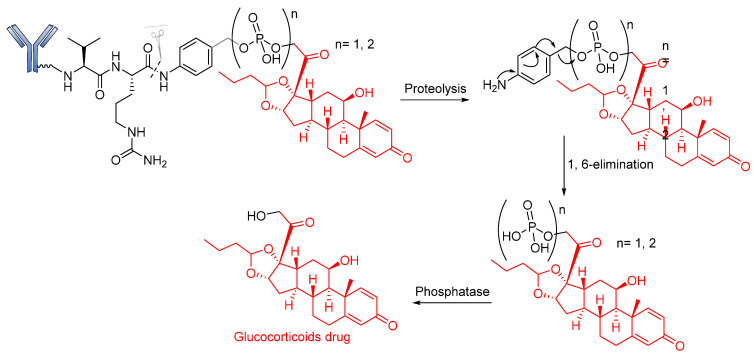
The mechanism by which an ADC containing pyrophosphate is released.

These belong to another vital class of enzyme-cleavable linkers expressed exclusively to target enzymes in the lysosomal compartment. These linkers target pyrophosphatase and acid phosphatase enzymes, which hydrolyze pyrophosphates and terminal monophosphates into their respective alcohols. Kern and coworkers reported the usefulness of a phosphate-bridged Cathepsin B-sensitive linker in delivering glucocorticoids to tumor cells [101]. By using the PABC spacer molecule, they developed an aqueous, soluble phosphate drug conjugate comprising the Val–Cit-PABC cleavable linker. Upon proteolytic cleavage of the dipeptide linker (Val–Cit), PABC is self-eliminated, leading to the hydrolysis of the terminal phosphate by phosphatase to release the payload (Figure 14). An in vitro study of ADCs containing this linker revealed that they showed high blood stability, rapid lysosomal cleavage, and aqueous solubility. While these properties thus support the applicability of this linker in bioconjugation and ADCs containing lipophilic payloads, there is no in vivo proof to validate this approach. Moreover, this linker system increases the space of ADC payload options by exploiting the attachment of a payload via the aliphatic alcohol of the phosphate group.

**Figure 15:**
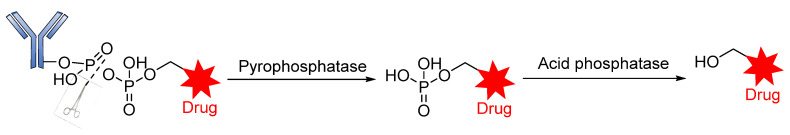
The mechanism by which an ADC containing pyrophosphate is released.

Recently, in similar but more elaborate research, Kern and coworkers reported the synthesis of highly soluble pyrophosphate-containing ADCs for the targeted release of glucocorticoids to immune cells [54,101]. By exploiting the biorthogonal property of the linkers, a phosphate diester was introduced into an alkyl chain. The drug conjugates underwent dual-enzymatic cleavage of pyrophosphatase and acid pyrophosphate to release the glucocorticoid (Figure 15). Nevertheless, as a proof of concept, it is significant for this linker to have been proven beyond merely in vitro laboratory analysis, using α-hCD70 and glucocorticoids as the delivery vehicle and payloads, respectively.

### 3.2. Non-Cleavable Linkers

**Figure 16:**
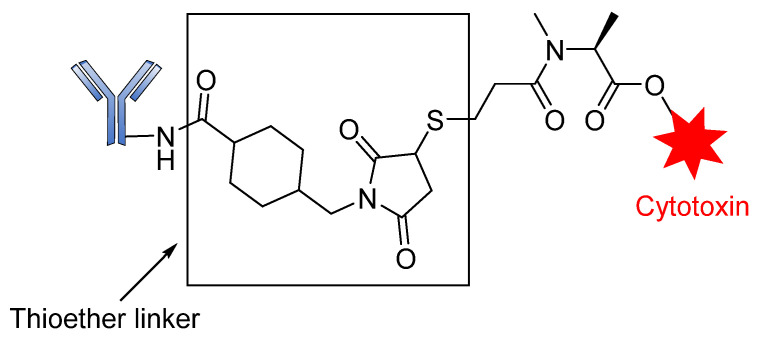
Structure of T-DM1 containing a thioether linker.

Non-cleavable linkers are divided into two groups, namely thioether or maleimidocaproyl (MC). They consist of stable bonds that prevent proteolytic cleavage and ensure greater plasma stability than their cleavable counterparts. ADCs containing this type of linker depend on the complete lysosomal enzymatic degradation of the antibody to release payloads after internalization, resulting in the simultaneous detachment of the linker [93,102]. This linker strategy has been successfully explored by Genentech/Immunogen, with clinical approval of Trastuzumab emtansine (Kadcyla/T-DM1). This ADC contains a non-cleavable SMCC (*N*-succinimidyl-4-(maleimidomethyl) cyclohexane-1-carboxylate) linker connecting a warhead DM1 cytotoxin to Lys residues of anti-HER2 mAb Trastuzumab (Figure 16). This drug conjugate displayed greater activity than the conventional Trastuzumab-DM1, or Trastuzumab conjugated to other maytansinoids via reducible disulfide linkers [56]. Similarly, Monomethyl auristatin F (MMAF) drug conjugates with non-reducible thioether linkers were found to be more stable than Val–Cit conjugates and they also preserved their potency [51]. Of note, non-cleavable linkers allow for the alteration of the chemical properties of the small molecule in order to tune affinity for the transporter or improve potency [51,103]. The comparative advantage of non-cleavable linkers over their cleavable counterparts is their increased plasma stability [104]. Overall, non-cleavable linkers offer a greater therapeutic window than cleavable linkers since the payload derivative from non-cleavable ADCs can kill the target cells [51,103].

### 3.3. Conjugation Strategies

The success of an ADC depends mainly on the conjugation strategy. Two main types of conjugation are used in ADC design, namely chemical and enzymatic approaches. Most ADCs exploit the presence of Lys and Cys residues at the junction sites of the antibody, which can be modified for directional coupling. A typical IgG_1_ antibody molecule has roughly 90 Lys residues, of which approximately 30 can be modified for conjugation, implying that between 1 and 30 payloads can be covalently coupled to the antibody. The amine group of Lys and sulfhydryl of Cys are used for the chemical conjugation of the antibody-linker [77].

**Figure 17:**
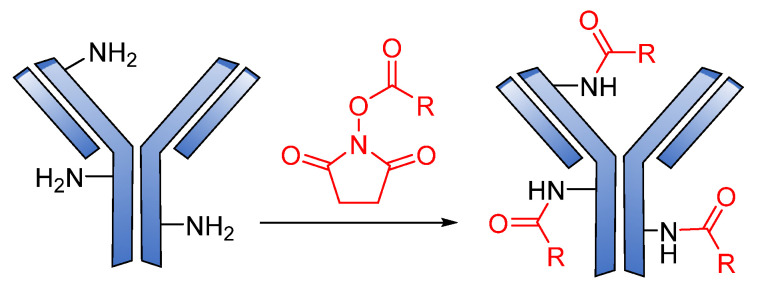
The mechanism for the formation of amide attachment sites through *N*-hydroxysuccinimide (NHS).

Generally, amide coupling is the method of choice for the chemical conjugation of payload and antibody Lys residues using activated carboxylic acid esters as linkers. This type of coupling gives a high-yielding ADC. The primary amine in Lys easily reacts with *N*-hydroxysuccinimide (NHS) esters introduced into the drug-linker, forming a stable amide, and a great number of commercial linkers rely on this method (Figure 17) [16,105].

**Figure 18:**
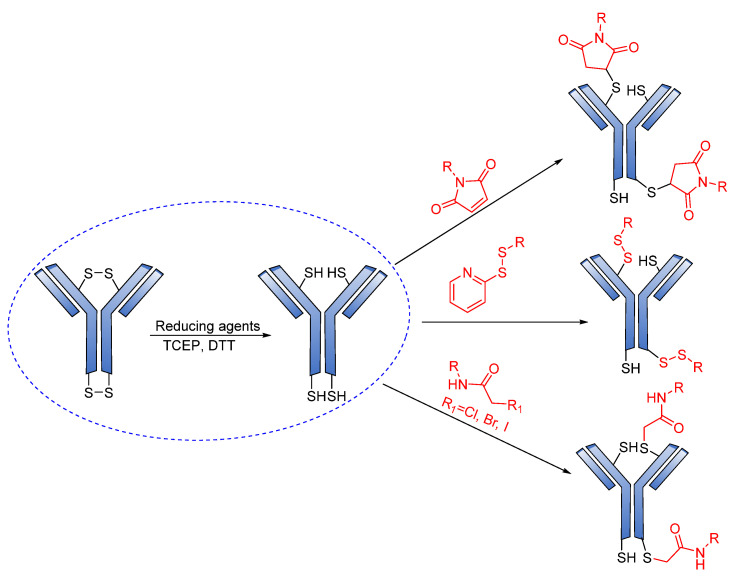
Mechanism for the formation of the sulfyhydryl attachment site.

Cys are present in the antibodies and form disulfide bridges. Under careful reduction conditions, the disulfide bridge can be reduced by tris(2-carboxyethyl) phosphine (TCEP) or DL-dithiothreitol (DTT) to afford reactive thiol groups; meanwhile, intrachain disulfide bonds retain their unique state. The free thiol groups as attachment sites on the antibodies are then conjugated with a small linker molecule through various chemical reactions, such as Michael additions, disulfide formation, and a-halo carbonyl alkylations (Figure 18) [105].

**Figure 19:**
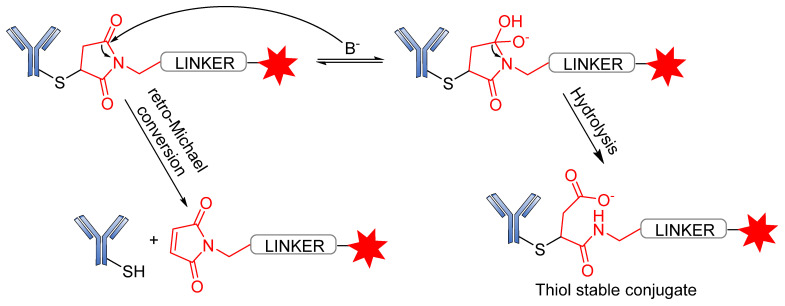
Side-reactions undergone by the succinimide–thioether moiety.

**Figure 20:**
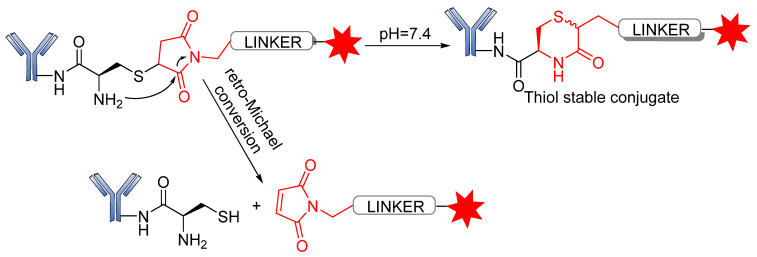
Mechanism for the locking of the thioether conjugation bond via transcyclization.

Several reports have revealed that a major challenge with the use of the maleimide-based strategy is its susceptibility to the premature release of payload through the retro–Michael reaction in the presence of blood thiols [105]. However, another reaction associated with the succinimide–thioether rings is hydrolysis, which converts the succinimide–thioether into succinic acid. This second side-reaction could help to prevent the retro-Michael reaction by firmly binding the conjugate to be thiol-stable (Figure 19) [106,107,108]. A new strategy recently developed by Lahnsteiner and coworkers to overcome the retro-Michael exchange is the formation of a stable 6-membered ring via transcyclization of the succinimide–thioether ring (Figure 20) [109].

## 4. Approved ADCs and ADCs in Clinical Trials

The importance of ADCs for cancer treatment is exemplified by the increasing number of these drugs on the market and in clinical studies for the treatment of solid tumors and hematologic malignancies [33,110]. There are currently 12 approved ADCs on the market (Table 2); however, considerable research efforts into the development of new ADCs are ongoing. Over 80 ADCs are now in clinical trials, and several others have been terminated due to linker instability and/or toxicity issues, among other factors [40,111]. The elements of approved ADCs are detailed in Table 2, while Table 3 covers selected examples of the ADCs in late-stage clinical trials.

**Figure 21:**
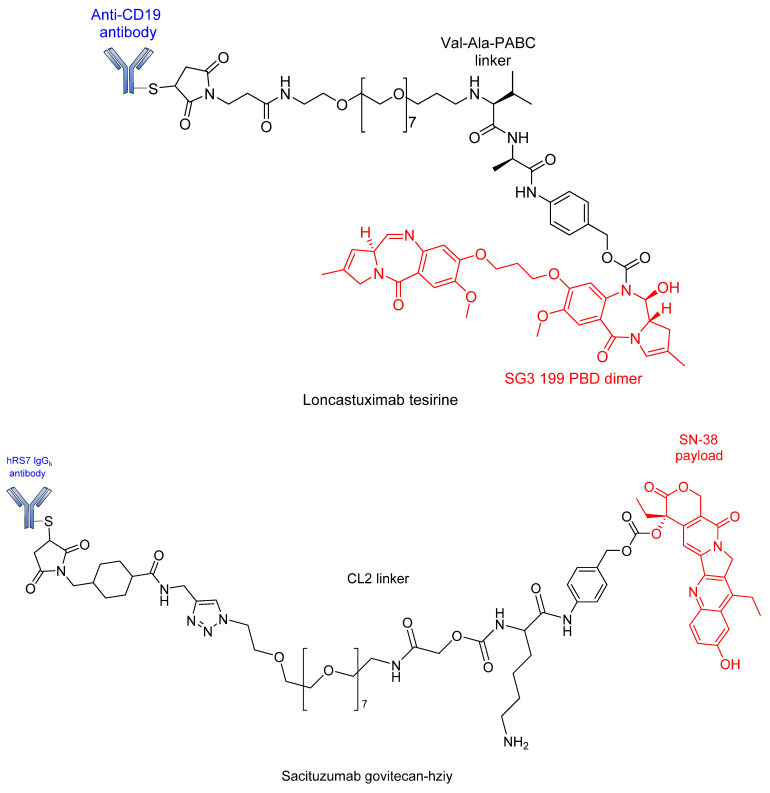

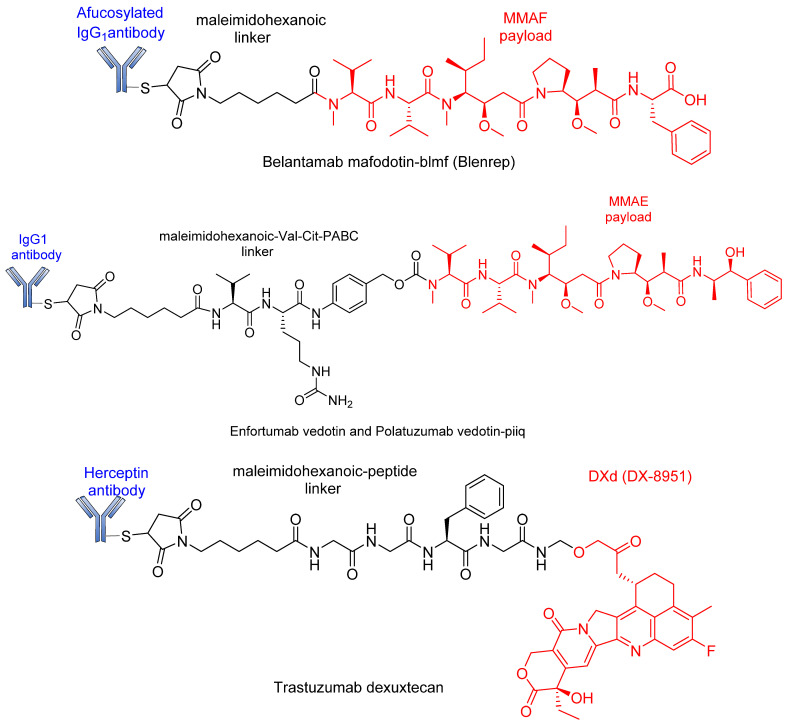
Chemical structures of some recently approved ADCs.

Figure 21 shows chemical structures of some recently approved ADCs.

## 5. ADC Mechanism of Action

**Figure 22:**
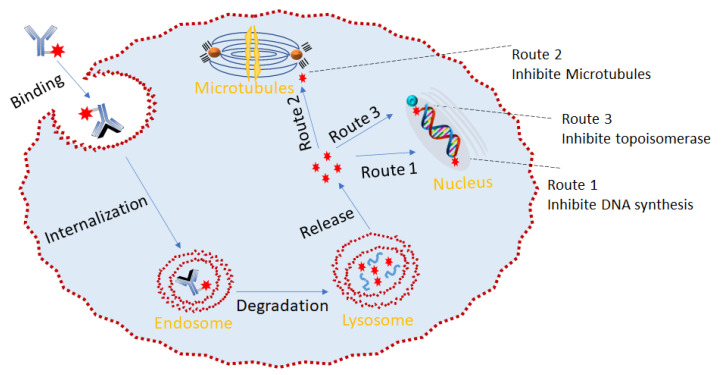
Mechanism of action of ADCs: ADC binds to a cell-surface antigen that is ideally specific to a cancer cell. Upon binding, the ADC-antigen is internalized into the tumor cell. When the complex is degraded, it releases the cytotoxin, which then binds to its target to cause cancer cell apoptosis.

The mechanism of action of the ADC is shown in Figure 22. Relying on its ability to specifically recognize a well-expressed antigen, the ADC acts similarly to a shuttle that selectively delivers cytotoxic agents into the tumor cell via receptor-mediated endocytosis. Upon effective internalization of the ADC-antigen complex, it is fused with the endosome, which cleaves the complex, leading to the simultaneous recycling of the antigen and transport of the ADC to the lysosome. The ADC then undergoes lysosomal degradation to release the cytotoxin. This cytotoxin then binds to its target, leading to apoptosis or cell death via either DNA intercalation (route 1 and route 3) or binding to microtubulins (route 2) (Figure 13) [46]. Due to the complexity of the ADC internalization process, the localization of the tumor antigen on the cell surface is highly relevant to achieve efficient ADC binding [158].

## 6. Conclusions and Prospects

Ten ADCs have been approved by the FDA in the last five years (Gemtuzumab ozogamicin was first approved in 2000, withdrawn in 2010, and reapproved in 2017), which means that approximately 4% of all drugs approved during these years (240 drugs have been approved in total) are ADCs. However, the importance of ADCs in the context of the current toolbox to treat diseases is best exemplified by the large number that are in clinical phases. This pipeline assures that many more ADCs will be approved in the coming years, thereby fueling research in this field. In this regard, special attention must be given to the linker used. A suitable linker remains the mainstay of a successful ADC. In this context, a linker must remain stable in circulation and guarantee the safe release of the payload in the cell (such as release by restriction endocluease in lysosome or release after antibody degradation). Chemically cleavable (hydrazone, disulfide) linkers, enzymatically cleavable (peptide-based, β-glucuronide-based) linkers, and non-cleavable (thioether, maleimido caproyl) linkers are currently those most commonly used in ADC design. Given that linkers generally influence the stability, toxicity, pharmacokinetic properties, and pharmacodynamics of ADCs, considerable care must be taken in their selection for ADC design. In addition, the linker must take into consideration the reactive groups on the cytotoxic drugs, including the mAb and derivative functional groups.

Most ADCs in clinical stages use the common Lys and Cys residue motifs for their conjugation. In this context, researchers are currently directing significant effort toward studying new linkers. For instance, photo-sensitive ADC linkers and biorthogonal cleavable linkers are emerging classes that are still under study [126]. Although these linkers have some advantages, such as specificity, potency, and low toxicity, ADCs containing them are yet to gain regulatory approval.

Despite the huge progress made in the development ADCs, it is still difficult to postulate the exact market size of these pharmaceutical agents in the near future. However, it is envisaged that the development of new linkers will enlarge the design of new ADCs to further bolster the scope of oncology.

## Figures and Tables

**Table 1:** A selection of cleavable linkage types, their structures, cleavage conditions, cleavage products, and site of cleavage.

ADC Structure	Linkage Type	Cleavage Mechanism	Products Formed	Cleavage Site
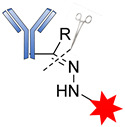	pH sensitive	Hydrolysis 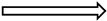	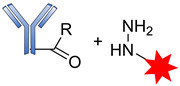	Lysosome/Endosome [57,58,59,60]
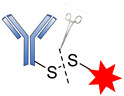	Reduction sensitive	Reduction 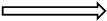	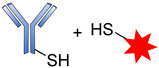	Cytoplasm [56,61,62,63]
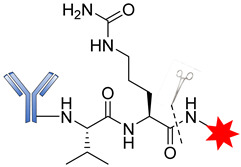	Peptide based linkage	Proteolysis 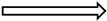	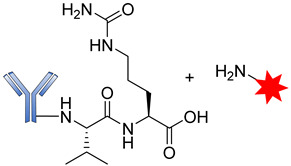	Lysosome [64,65,66,67]
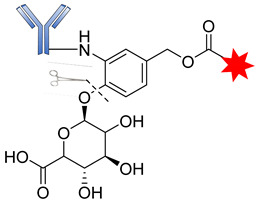	β-glucoronide	Glyosidase 1, 6-Elimination 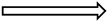	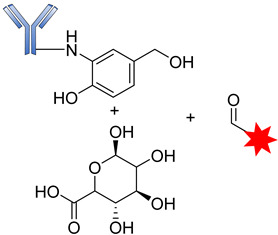	Lysosome [68,69]
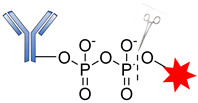	Phosphatase cleavage	Phosphatase 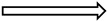	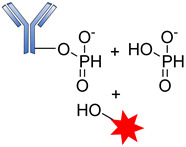	Lysosome [70]
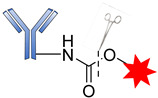	Carbamate	Esterase 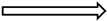	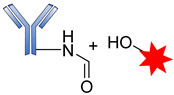	Lysosome [71,72]
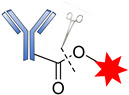	Ester	Esterase 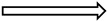	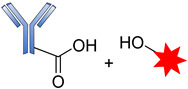	Lysosome [72,73]

**Table 2:** Antibody-drug conjugates approved.

API	Trade Name	Developer	mAb	Linker	Cytotoxin	Target Antigen	Indication(s)	Phase
Gemtuzumab ozogamicin	Mylotarg	Pfizer	Hp67.6 (Hz IgG4)	Hydrazone	Calicheamicin	CD33	Acute myeloid leukemia	Approved 2000 [112], withdrawn 2010 [113]; reapproved 2017 [19].
Brentuximab vedotin (SGN-35)	Adcetris	Millennim/Takeda/Seattle Genetics	cAC10 (SGN-30, Ch-IgG1)	Dipeptide (VC)	MMAE	CD30	Hodgkin lymphoma, systemic anaplastic large cell lymphoma	Accelerated approval 2011 [87]; full approval 2015 [114].
Trastuzumab emtansine	Kadcyla	Roche/Genetech	Trastuzumab (Hz IgG1)	Non-cleavable (SMCC)	DM1	HER2	HER2-positive breast cancer	Approved 2013 [115,116]
Inotuzumab ozogamicin	Besponsa	Pfizer	G5/44 (Hz IgG4)	Hydrazone	Calicheamicin	CD22	Acute lymphoblastic leukemia	Approved 2017 [117,118]
Moxetumomab pasudotox-tdfk	Lumoxiti	AstraZeneca	Anti-CD22	Hydrazone	Pasudotox-tdfk	CD22	Relapsed hairy cell leukemia	Approved 2018 [119,120]
Polatuzumab vedotin (RG7596, TAB-897, DCDS4501A	Polivy	Genentech/Roche	Anti-CD79b (Hz IgG1)	Dipeptide (VC)	MMAE	CD79b	Relapsed or refractory diffuse large B-cell lymphoma	Approved 2019 [19,121,122]
Enfortumab vedotin	Padcev	Agensys/Astellas	Enfortumab	Dipeptide (VC)	MMAE	Nectin4	Solid and urothelial tumors	Approved 2019 [122,123,124]
Trastuzumab deruxtecan	Enhertu	AstraZeneca/Daiichi Sankyo	Trastuzumab (Herceptin)	Non-cleavable (mc)	Deruxtecan	HER2	HER2-positive breast cancer	Approved 2019 [125,126]
Sacituzumab govitecan	Trodelvy	Immunomedics	hRS7 IgGk	Acid-labile ester	SN-38	Trop-2	Triple-negative breast cancer, urothelial and other cancers	Approved May 2020 [127,128,129,130,131,132]
Belantamab mafodotin-blmf	Blenrep	GlaxoSmithKline (GSK)	IgG1	Non-cleavable (mc)	MMAE	BCMA	Multiple myeloma	Approved 2020 [132,133,134,135,136]
Loncastuximab tesirine-lpyl	Zynlonta	ADC Therapeutics	Anti-CD19	Dipeptide (VA)	PBD	CD19	Large B-cell lymphoma	Approved 2021 [137,138]
Tisotumab vedotin tftv	Tivdak	Seagen Inc	Tisotumab	Dipeptide (VC)	MMAE	Tissue factor	Metastatic cervical cancer	Approved 2021 [139,140]

**Table 3:** Selected ADCs currently in clinical trials.

ADC Name	Developer	mAb	Linker	Cytotoxin	Target Antigen	Indication(s)	Phase
Rovalpituzumab tesirine	Sanofi/ImmunoGen	Anti-DLL3 (Rovalpituzumab)	Dipeptide (VC)	PBD dimer	DLL3	Small-cell lung cancer	III [141,142,143]
Glembatumumab vedotin	Seattle Genetics/Celldex/Progenics	CR-011 (Hu IgG2)	Cleavable dipeptide	MMAE	gpNMB	Metastatic breast cancer and melanoma	II/III [144,145]
PSMA ADC	Seattle Genetics/Progenics	Anti-PSMA (Hu IgG1)	Cleavable dipeptide	MMAE	PSMA	Prostate cancer	II [121]
Pinatuzumab vedotin	Roche/Genentech	Anti-CD22 (Hz IgG1)	Cleavable dipeptide	MMAE	CD22	Diffuse large B-cell lymphoma, follicular non-Hodgkin lymphoma	II [121]
Telisotuzumab vedotin	AbbVie/Pierre Fabre	ABT-700	Cleavable dipeptide	MMAE	ABT-700	Advanced solid tumors cancer and non-small cell lung cancer	II [146,147]
Ladiratuzumab vedotin SGN-LIV1A	Seattle Genetics	Anti-LIV1 (Hz IgG1)	Cleavable dipeptide	MMAE	LIV-1	Breast cancer, lung cancer	II [148]
Mirvetuximab soravtansine	ImmunoGen	M9346A	Cleavable disulfide	DM4	FOLR1	Ovarian, endometrial, non-small cell lung cancer	III [149,150]
Lorvotuzumab mertansine	ImmunoGen	huN901 (Hz IgG1)	Cleavable disulfide	DM1	CD56	Leukemia	II [88]
Coltuximab ravtansine	ImmunoGen	huB4 (Hz IgG1)	Cleavable disulfide	DM4	CD19	Diffuse large B cell lymphoma, acute lymphocytic leukaemia	II [93,151,152]
Indatuximab ravtansine	Biotest/ImmunoGen	Nbt062, Anti- CD138 (Ch IgG4)	Cleavable disulfide	DM4	CD138	Multiple myeloma	II [153]
Anetumab ravtansine	Bayer Health Care	Antimesothelin (Hz IgG1)	Cleavable disulfide	DM4	Mesothelin	Mesothelioma and other solid tumors	II [115,154]
SAR566658	Sanofi	DS6 (Hu IgG1)	Cleavable disulfide	DM4	CA6	Triple-negative breast cancer	II [155,156]
Depatuxizumab mafodotin	AbbVie	ABT-806	Non-cleavable (mc)	MMAF	EGFR	Glioblastoma and other EGFR-positive tumors	III [129]
Naratuximab emtansine	ImmunoGen	K7153A humanized IgG1	Non-cleavable (SMCC)	DM1	CD37	Diffuse large B cell lymphoma and follicular lymphoma	II [121]
AGS-16C3F	Agensys/Astellas	Anti-AGS16 (Hu IgG2a)	Non-cleavable (mc)	ENPP3	ENPP3	Renal cell carcinoma	II [157]

## Data Availability

Not applicable.
